# Machine Learning in Interpolation and Extrapolation for Nanophotonic Inverse Design

**DOI:** 10.1021/acsomega.2c04526

**Published:** 2022-09-09

**Authors:** Didulani Acharige, Eric Johlin

**Affiliations:** Department of Mechanical and Materials Engineering, Western University, London, Ontario N6A 5B9, Canada

## Abstract

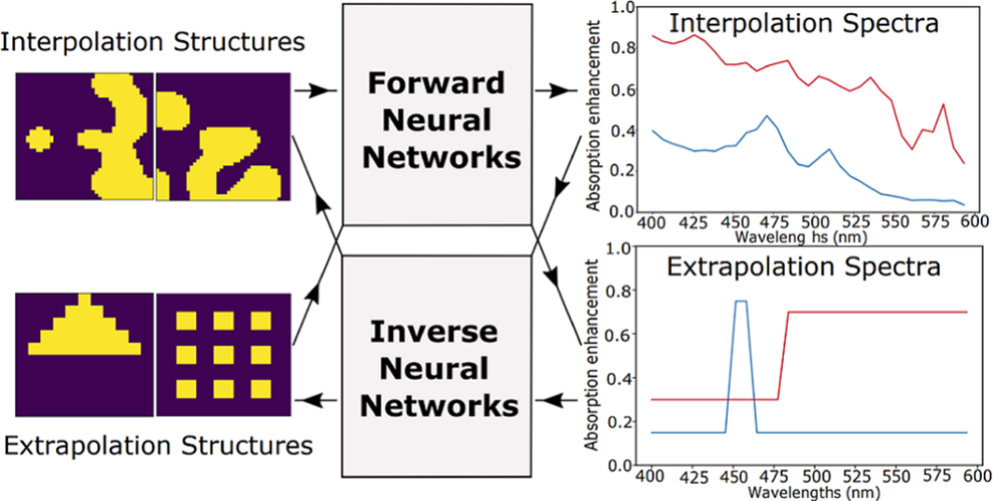

The algorithmic design of nanophotonic structures promises to significantly improve the efficiency of nanophotonic components due to the strong dependence of electromagnetic function on geometry and the unintuitive connection between structure and response. Such approaches, however, can be highly computationally intensive and do not ensure a globally optimal solution. Recent theoretical results suggest that machine learning techniques could address these issues as they are capable of modeling the response of nanophotonic structures at orders of magnitude lower time per result. In this work, we explore the utilization of artificial neural network (ANN) techniques to improve the algorithmic design of simple absorbing nanophotonic structures. We show that different approaches show various aptitudes in interpolation versus extrapolation, as well as peak performances versus consistency. Combining ANNs with classical machine learning techniques can outperform some standard ANN techniques for forward design, both in terms of training speed and accuracy in interpolation, but extrapolative performance can suffer. Networks pretrained on general image classification perform well in predicting optical responses of both interpolative and extrapolative structures, with very little additional training time required. Furthermore, we show that traditional deep neural networks are able to perform significantly better in extrapolation than more complicated architectures using convolutional or autoencoder layers. Finally, we show that such networks are able to perform extrapolation tasks in structure generation to produce structures with spectral responses significantly outside those of the structures on which they are trained.

## Introduction

Nanophotonics has seen significant growth over the past 2 decades, with increasing interest in discovering new physics and technologies that utilize interactions between light and materials at the nanoscale.^1^ Algorithmic design of nanophotonic structures promises to significantly improve the efficiency of nanophotonic components due to the strong dependence of electromagnetic function on geometry. However, due to the wave nature of light, the structure of such components can be highly nonintuitive, limiting the effectiveness of traditional design approaches. Therefore, several approaches have been introduced to improve the design of nanophotonic structures, including genetic algorithms^2,3^ and adjoint methods,^4,5^ among other inverse design algorithms.^6^

While these conventional algorithmic design approaches have shown great improvements over traditional manual design, their response is fully dependent on the input conditions, with little intuitive correlation between the initial conditions and constraints on the design problem. This generally means that components need to be fully redesigned when any parameters or constraints are modified. This can make the use of such design approaches, particularly for complex structures, become highly time-intensive and computationally expensive due to the immense number of possible combinations of features.^1^ In particular, when 3D or reconfigurable new geometries are desired, it requires fully configuring the topology of a structure and this is computationally expensive as it fully depends on the computational simulations. Moreover, these numerical simulations are not assured to find globally optimal solutions, and often, a large number of initial conditions must be tested to map out the configuration space, even when single designs are desired.^5^

Recent theoretical results have shown that data-driven machine learning (ML) techniques in nanophotonic design have the promise of reducing some of this high computational cost.^7−10^ The potential behind ML, and in particular artificial neural network (ANN) techniques, is that such algorithms can be used to improve the design even without directly solving Maxwell’s equations. This allows a trained system to be able to either analyze or generate structures without needing time-intensive numerical simulations. Different ML and ANN techniques,^11^ such as convolutional neural networks (CNNs),^12,13^ deep neural networks (DNNs),^14,15^ and generative adversarial networks (GANs)^16,17^ have been explored for the forward^18−20^ and inverse design^7,14,16,21−26^ of nanophotonic structures. Moreover, recent works have investigated the use of pretrained networks^27−29^ in photonic applications, showing the promise of transfer learning as well.

There is considerable interest in generating high-performing nano-optical structures, especially structures that can express responses outside of the trained configuration space. While previous work on nanophotonics has begun to investigate this potential, the explorations have been limited. These generally involve either specifically limiting to one type of spectrum (e.g., Lorentzian line shapes^17,30^) or limiting the scope of the modifications to perturbations of the explored configuration space.^31^

There is still much debate in the ML literature as to the extent that extrapolation is possible, particularly in generative design. Also, while the performance of variations on specific network architectures has been investigated (e.g., modifications on GANs^32^ and different types of pretrained networks^28^), comparison across vastly different types of ML approaches for nanophotonic structure generation is under-explored.

Herein, we work to address both of these limitations. We first explore the ability of both interpolation and extrapolation of optical responses to determine the capability and limitations of a variety of prototypical ML approaches to generating structures both within and outside of the trained configuration space. Furthermore, by utilizing probability density functions of the errors of generated solutions, we are able to distinguish between networks that show high but unreliable performance and those with more moderate but consistent predictions, either of which may be preferential depending on a specific application’s needs. Additionally, we investigate combining different classical ML approaches [such as principal component analysis (PCA)] with ANN techniques as combining vastly different ML approaches provides the opportunity for the strength of one network to balance out the weakness of the other.^33−35^

This exploration leads to three main contributions: first, the separate analysis of both interpolation and extrapolation capability of different networks shows that different architectures indeed show different aptitudes for each of the two types of forward or generative prediction, as well as different levels of performance and reliability. Second, through the examination of networks pretrained on general image classification, we show that strong performance can be achieved with significantly reduced training times. This should prove valuable for particular situations with limited amounts of data, training under limited computational and time conditions, to train networks required to performing well in versatile use cases, and so forth. Finally, we find that DNNs are in particular able to perform better in extrapolative generation than more complicated networks, such as convolutional networks, and are able to generate structures with responses significantly outside the phase space on which they are trained. This indicates great promise for the use of ANNs generally to design structures with features or levels of performance surpassing their training set.

## Methods

In this study, we explore the use of both forward and inverse ML networks for the algorithmic design of nanophotonic structures. We compare a variety of network types and architectures in their performance both in interpolation and extrapolation to find the most accurate and efficient networks and architectures to generate desired structures.

### Training Set

**Figure 1:**
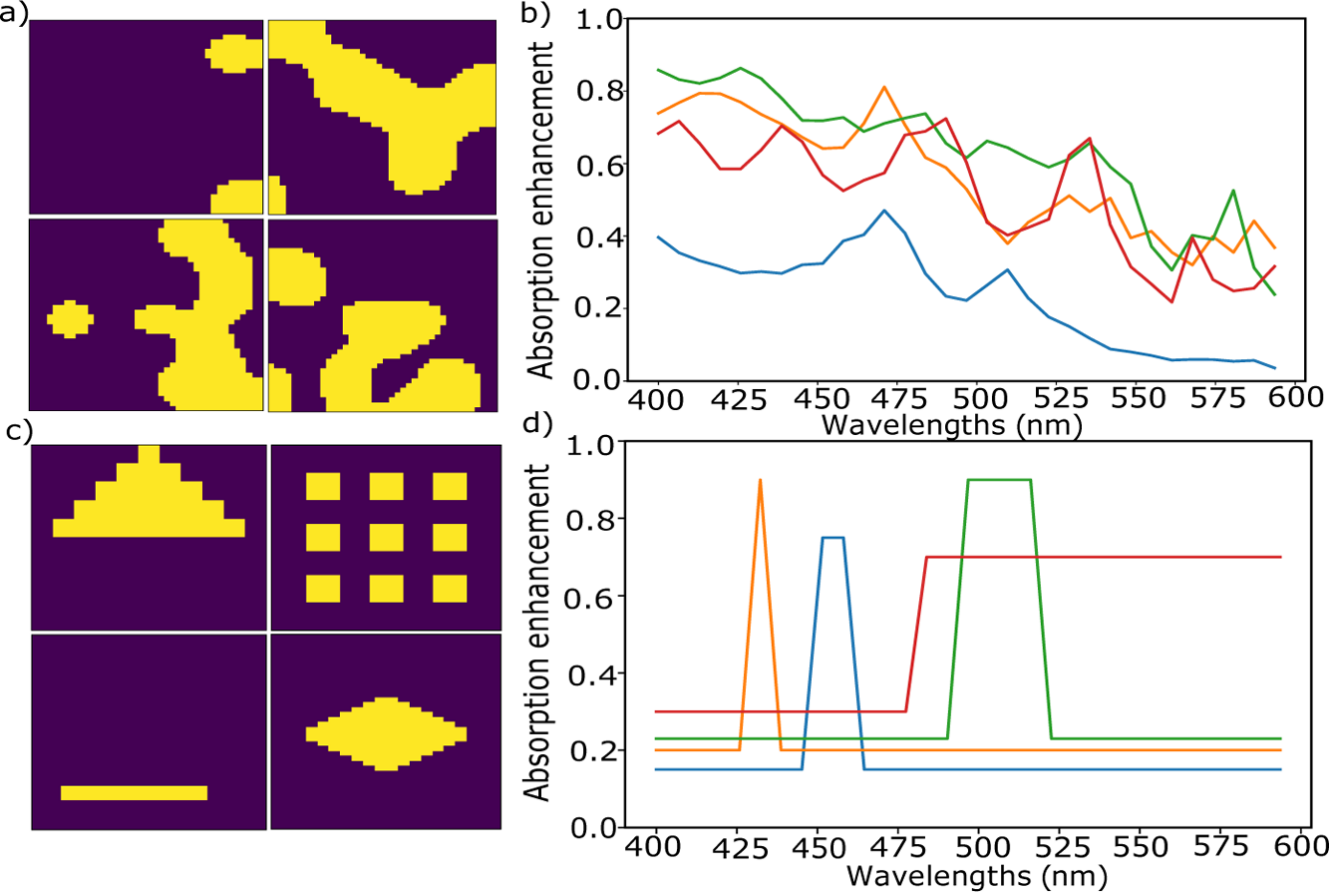
Examples of the interpolation and extrapolation structures and spectra. Interpolation structures (a) and corresponding spectra (b), which are used to train forward and inverse networks. (c) Extrapolation structures that are used to test forward networks. (d) Extrapolation spectra that are used to test inverse networks.

In order to provide a training set for our exploration of ML in nanophotonics, we investigate a conceptually simple model system with a diverse range of optical responses—specifically silicon nanoparticles with overall sizes bound by a 500 nm square cross-sectional area. We consider the response of these nanoparticles in terms of absorption enhancement for visible wavelengths, ranging from 400 to 600 nm (Figure 1b). This size–wavelength–material combination supports the exploration of optical resonances, interference effects, as well as refraction, reflection, and transmission of the incident light, all leading to a variety of spectral responses in terms of absorption enhancement or degradation. The peaks and troughs observed in optical responses are likely due to cavity effects within the structures. These result from parallel faces arising from the use of rectangular regions added randomly to the structures during data generation. These agree qualitatively with the responses seen in similar computational structures in the literature.^28^

Each nanoparticle in this work is defined by a 40 × 40 matrix, defining the presence or absence of silicon, formed through the random addition of boxes of random size and quantity to the matrix, and then smoothed (Figure 1a). An ensemble of 10,000 such structures is simulated in 2D using the open-source finite-difference time-domain (FDTD) simulation software MEEP.^36^ Simulations are terminated when fields decay by a factor of 10^–6^. The simulation conditions are tested for convergence across a range of representative samples, with plots demonstrating overall convergence with respect to the simulation resolution and boundary conditions shown in the Supporting Information, Figure S2.

The absorption enhancement spectra were treated as labels, while the structures were considered as input data to feed into the forward neural networks. Four different types of networks were implemented to obtain optical responses from the structures, while five different inverse networks have been implemented to generate new structures.

The concept of extrapolation has been an interesting topic in the ML community, and the idea of extrapolation in this context is to predict the absorption enhancement values corresponding to structures significantly outside of the configuration space or to generate structures corresponding to absorption enhancement spectra outside of the training data set. Figure 1 represents some of the interpolation structures and spectra along with extrapolation structures used as inputs for forward networks, and extrapolation spectra used as inputs for inverse networks. The calculated minimum structural errors (binary cross-entropy error) between the training and validation and the training and extrapolation samples are 0.017 and 1.679, respectively. Therefore, the extrapolation structural error is 98 times higher compared to the validation error with the training dataset. This indicates that extrapolation structures are comparatively different from interpolation samples.

It should be noted that the extrapolation spectra in Figure 1d are unrelated to the extrapolation structures in Figure 1c and represent idealized performance that may not be theoretically possible to fully achieve. The goal of these structures is to push the network to generate as close as possible to the performance of the novel spectrum, thereby allowing a measurement of the degree to which extrapolation is possible.

### Forward Models

**Figure 2:**
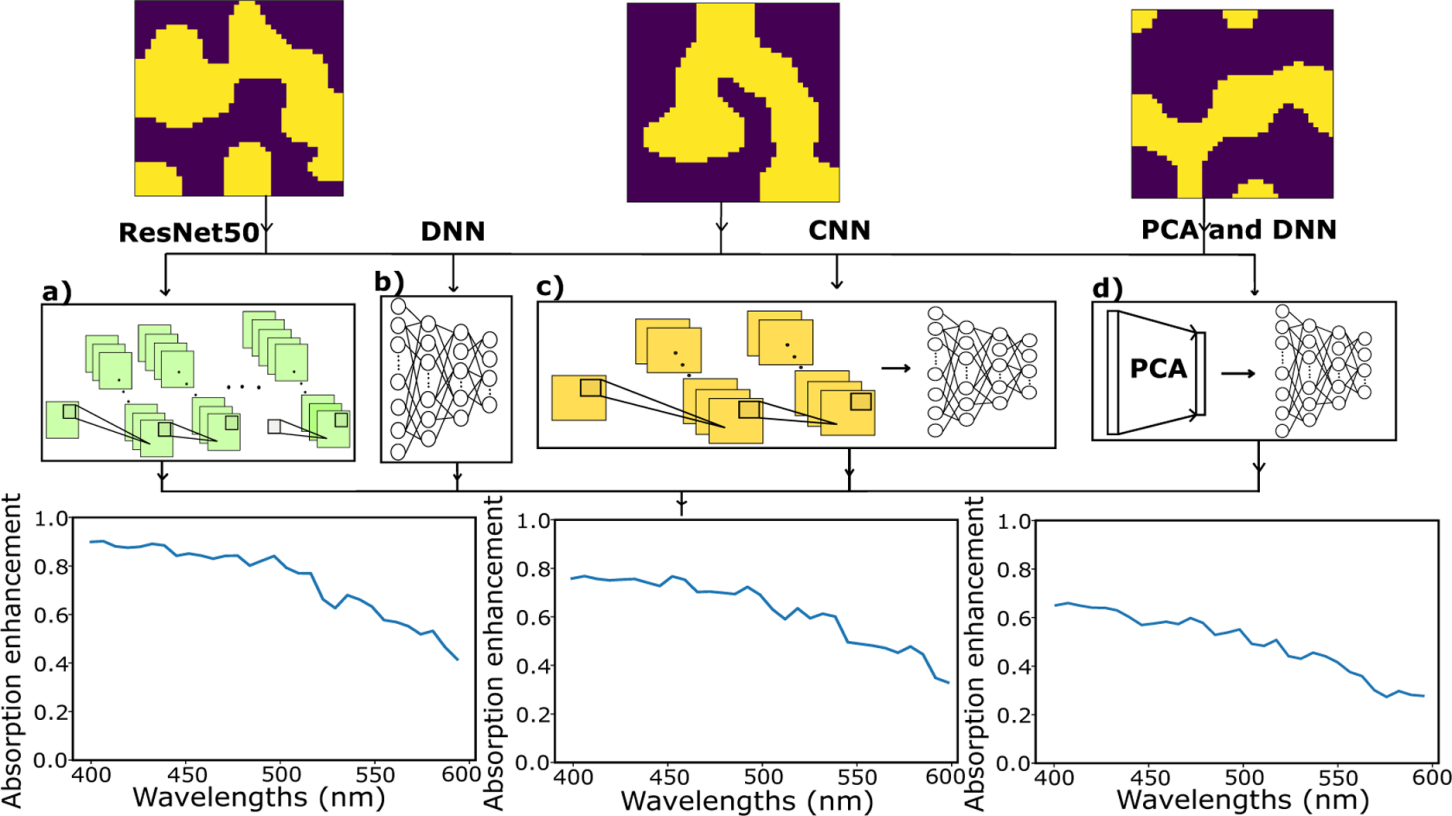
Schematic representations of the four different networks implemented for forward design. (a) ResNet50 pretrained network, (b) DNN (c) CNN, and (d) combined approach of PCA and DNN. Sample inputs from the 500 nm × 500 nm square silicon nanoparticle training set are shown above, with yellow color representing the presence of silicon and purple indicating the absence. Example spectra from the corresponding model and sample are shown below the network.

The goal of forward modeling is to implement a network that can predict the absorption enhancement spectrum from an input structure. There are different ML and ANN approaches promising to find out the mapping between such inputs and the outputs. Conventional DNNs and deep networks with convolutional layers are two of the most prominent types of such networks, with the choice between the two often depending on the requirements of the learning problem. In this work, we explored both of these two types of neural networks as shown in Figure 2, with two specific variations of each. For the convolutional networks, a pretrained network, ResNet50, and a CNN which is fully trained from scratch to obtain the optical responses are considered. For the deep networks, a purely DNN, as well as DNN combined with a classical ML algorithm, PCA, are implemented. This allowed the comparison of purely ANNs with a combined ensemble network approach. The method of PCA has the ability to reduce the dimensionality^37^ of data keeping the most significant features. Details of the utilized networks can be found in the Supporting Information, with specific architectures available in Supporting Information Table S1.

### Inverse Models

**Figure 3:**
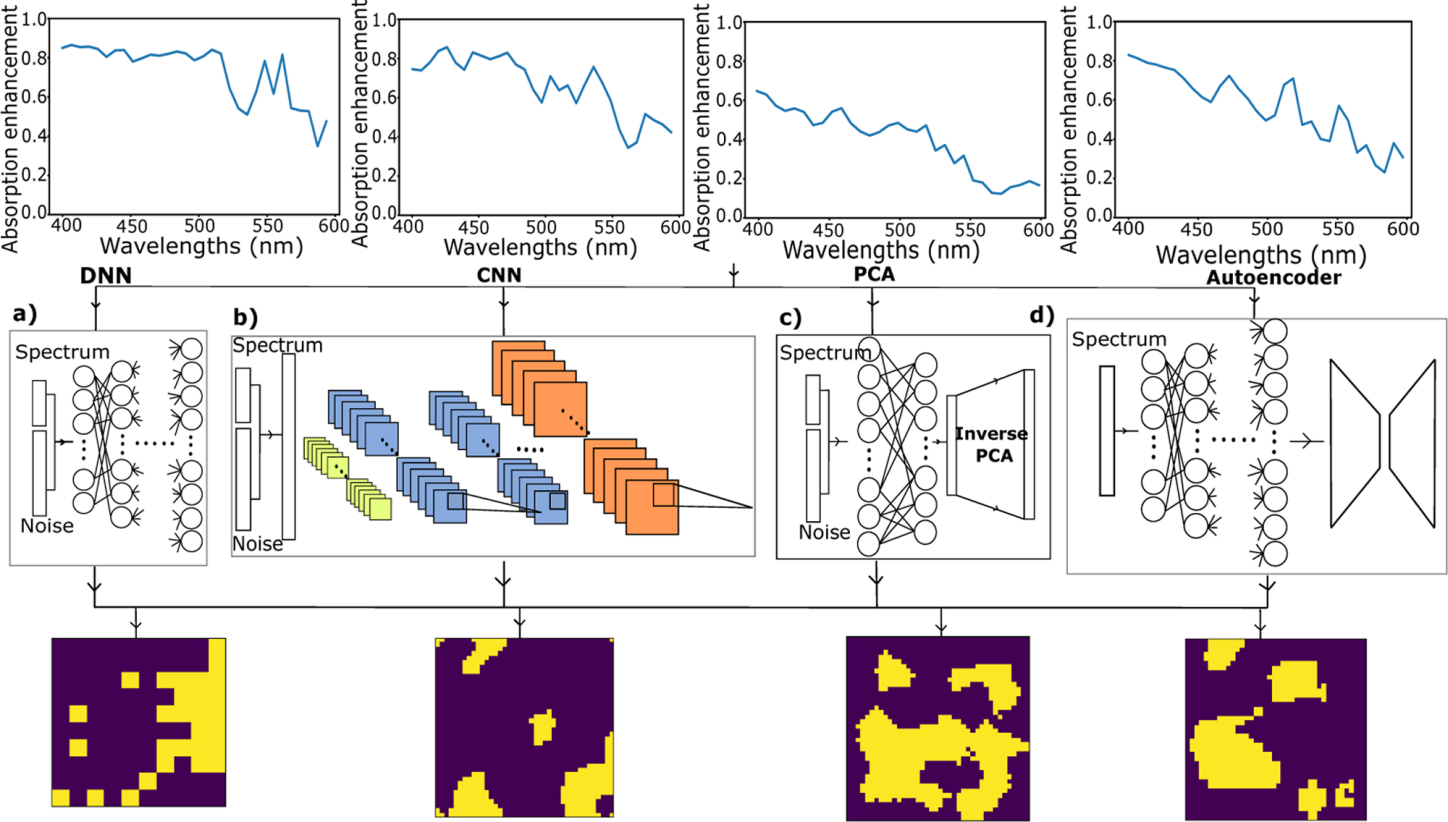
Four main different networks implemented for inverse design (a). A DNN (b). A CNN (c). A combined approach of PCA and DNN (d). An autoencoder and DNN. Four representative input spectra from the training set are shown above the schematics, with the corresponding structures generated by the respective networks shown below the schematics.

After implementing and training the forward networks, five different inverse networks were constructed to generate structures from desired input spectra to compare performances to find the best-performing inverse network. Two implementations of CNNs, a DNN, a CNN combined with an autoencoder, and an inverse PCA approach combined with a DNN were all employed, as shown in Figure 3.

As our first inverse network, we implemented a DNN to generate structures from the desired input spectra. A noise vector is additionally fed in as an input to the network with the purpose of allowing the network to generate different structures corresponding to the same spectral values. This is useful as it gives the network multiple chances to produce a well-performing structure for a particular input while also breaking the symmetry between similar spectra with vastly different structures in the training phase. As the second and third inverse networks, we implemented two CNNs with upsampling layers, one of which utilizes true label (spectra) values in training, while the other utilizes spectra predicted by the forward CNN from the label structure. In both CNNs, a noise vector is fed into the network to generate different spectra for similar absorption enhancement spectra.

In the fourth network, the structures generated from the CNN were encoded to a latent space and then fed to a decoder network to match the structures to the original structures. The purpose of the implemented autoencoder is to reduce excessive complexity and noise in the structures generated from the CNN. The loss comparison for the optimization of this network was done with the generated structures and the original structures from the training set. Finally, as a combined approach, an inverse PCA method was implemented by feeding the 500 components obtained from the DNN to the inverse PCA algorithm to generate structures. The PCA network that was previously trained during the forward modeling was used here, simply with a reversed propagation through the system.

### Model Training

All four forward networks were trained for 100 epochs and utilized early termination to ensure that the final network weights correspond to those with the lowest validation loss. The number of iterations to train inverse networks was decided based on their performances and the time taken to train each network. The hyperparameters used for inverse networks are given in Table S2 of the Supporting Information.

In order to benchmark the actual ability of these networks to generate structures with accurate spectral responses, upon completion of inverse network training, 20 spectra in both interpolation (i.e., spectra from the validation set, representing spectra similar to those the network was trained on but not in the training set), as well as extrapolation (highly idealized spectra with features vastly different from those in the training set), were run as inputs to the networks. Each of these input spectra was run with 10 different values of the noise vector to generate 10 different structure options for the input spectrum. It should be noted that this was not possible for the autoencoder network as it was unable to take a noise vector input and can thus only generate a single structure for any given input spectrum. The produced structures were then run in MEEP FDTD simulations to determine the true spectral response of the generated samples.

## Results and Discussion

To generate desired structures by improving the algorithmic design, we first implemented forward networks to predict the absorption enhancement. Afterward, we compared the performances of four different networks to find out the best-performing network and the architecture.

### Forward Modeling Results

**Figure 4:**
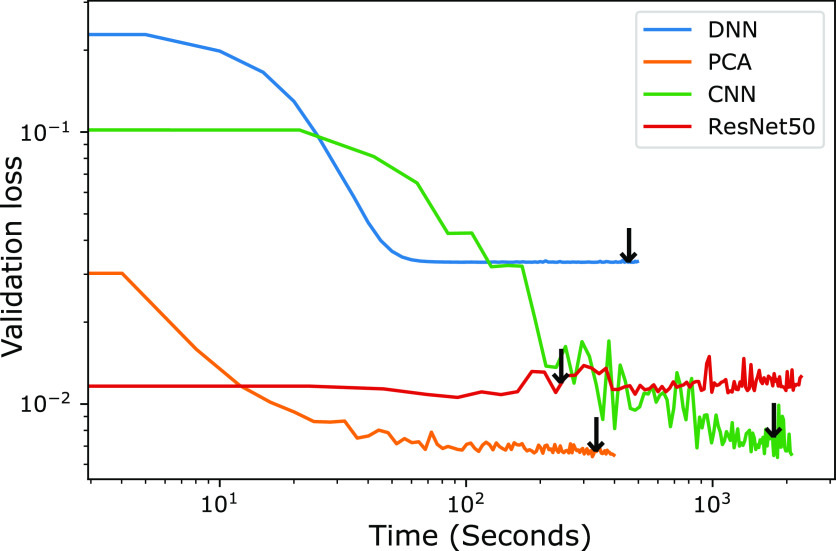
Validation loss variation of forward networks over training time for 100 epochs. Black arrows indicate the minimum validation loss location and thus the point of early termination.

We begin by exploring the performance of forward networks implemented to predict the optical response from input structures. Figure 4 shows a comparison of the validation loss with respect to training time for 100 epochs for the studied forward networks. The DNN implemented with the PCA approach takes the shortest time to reach the end of the 100 epoch cycle as the number of parameters of the network is fewest compared to other networks. This is mainly because PCA can reduce the dimensionality of the data by extracting the most dominant features, allowing a simplified DNN structure to be used. This additionally shows one of the lowest losses for the validation set, surpassed only (and very slightly) by the CNN. Although the training time for the DNN is lower compared to the CNN, the pure DNN shows the highest (worst) overall validation loss. This suggests that the network is unable to recognize and extract important features with a high number of inputs (1,600) and limited complexity of the network.

Early termination (convergence) allows for networks to use the weights for the lowest observed validation loss as opposed to the weights at the end of a fixed number of iterations. In Figure 4, we denote this point by the black arrows above each training line. While the full 100 epoch training time for the ResNet50 network is quite long, the convergence time is less than any of the networks, especially when compared to the CNN, showing the significant time savings of utilizing pretrained weights in such networks. The CNN takes a similar time to reach 100 epochs but by far the longest to converge. This is due to the more computationally expensive convolutional processes leading to a longer time per epoch (relative to DNNs) and lack of initial weights requiring nearly the full 100 epochs to converge (unlike ResNet50). However, while the CNN training is relatively slow, this network shows the lowest loss of any of the considered approaches and is thus expected to perform best in the task of prediction of optical behavior. While training times overall can be quite significant, one key advantage of using ML and ANN techniques is that once the networks are trained, all four networks take a fraction of a second to predict the spectra of a structure fed into the network.

**Figure 5:**
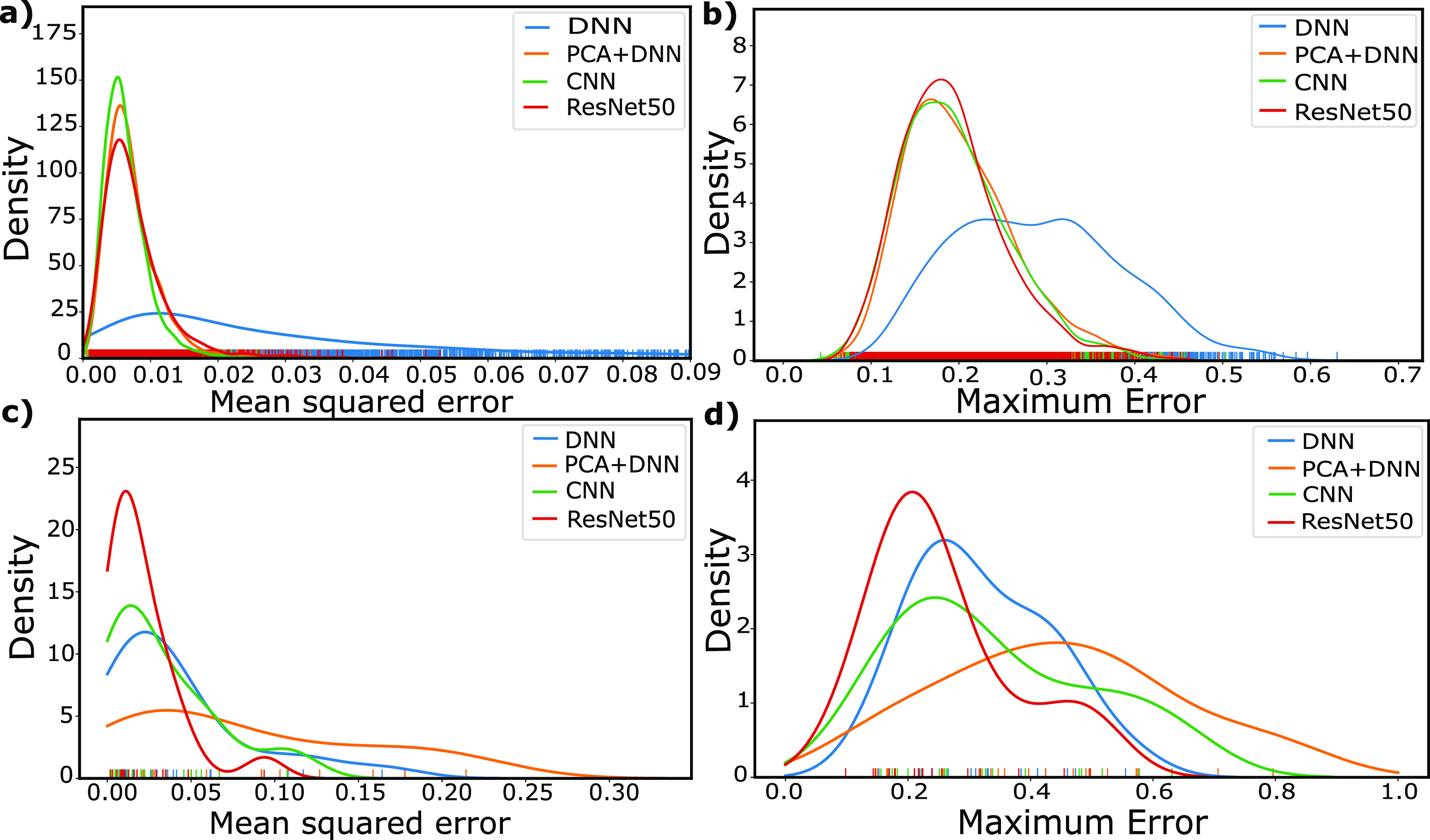
(a) Mean squared error variation for the validation set of interpolative structures. (b) Maximum absolute difference between peaks of true and predicted spectra of interpolative structures. (c) Mean squared error variation for the validation set of extrapolative structures. (d) Maximum absolute difference between peaks of true and predicted spectra of extrapolative structures. Color tick marks on the horizontal axis indicate the location of the included data points, allowing the range and density of samples to be visualized.

While the use of a validation set provides information on how the system will perform on average in interpolation, it does not indicate if all features in a spectrum are reproduced nor how the network will perform in extrapolation. To address these limitations, we further analyze the performance of the forward networks for both interpolation and extrapolation by using samples inside and outside the configuration space, testing the mean error of the predicted spectra, as well as the maximum error between the label spectra and predicted spectra. The maximum error is specifically investigated in order to determine if sharp peaks and troughs in the true label spectra are accurately reproduced. This is important as it indicates whether the systems are able to not only reproduce average responses but also anticipate resonant behavior. The results are interpreted through the visualization of probability density distributions as the error is often not normally distributed. For these distributions, an ideal system would show a delta-function peak at an error of 0, indicating that all samples have a perfect agreement with their label value. For realistic systems, a more strongly peaked response represents more consistent predictions, and proximity to 0 indicates a lower error. Our findings are depicted in Figure 5, testing both the mean spectral loss and peak/trough prediction capability of spectra, in interpolation (Figure 5a,b, respectively) and extrapolation (Figure 5c,d, respectively).

In interpolation response, the CNN, ResNet50, and PCA + DNN combined approach all perform fairly similarly, with a peak in their mean squared error (MSE) distribution near 0.005, with the CNN showing slightly dominant performance with a more strongly peaked distribution (Figure 5a). The pure DNN however performs relatively poorly, with an MSE peak at nearly twice that of the other networks, with similarly low performance and extended distribution of the maximum error (Figure 5b). This suggests that combined approaches indeed have the potential to perform better for interpolation as the combined approach of PCA + DNN shows significantly higher performance than the pure DNN.

When comparing the results of extrapolative structures, superior results are shown by ResNet50 both in terms of lower MSE (Figure 5c) and lower maximum error (Figure 5d), with more strongly peaked distributions at lower error values than any of the other systems. Therefore, ResNet50 appears to be the overall dominant performer, with similar performance in interpolation and significantly superior results in extrapolative structures. This is particularly interesting considering that this network had the shortest overall training time, suggesting that such pretrained networks even when pretrained on vastly different tasks (here, image recognition) can still perform extremely well. This is likely due to the diversity of data it was pretrained on, with the idea of using pretrained models being to take the advantage of the common features of the pretrained data set and our data set such as the importance of edges, circles, lines, and so forth.

While the combined approach of PCA + DNN performs quite well for interpolation, it performs by far the worst for extrapolation when compared to other networks, both in mean performance, as well as the ability to predict peaks and troughs. Interestingly, in these extrapolation samples, we now see an inversion of the trend in interpolation, with the combined PCA + DNN system performing worse than the pure DNN. This suggests that while the PCA step is able to reduce the sample complexity and thereby allow the DNN to predict spectra more accurately in interpolation, this leads to a type of overfitting, preventing the system from performing reasonably in extrapolation. Conversely, while the DNN does not work particularly well in interpolation, it has nearly identical performance in extrapolation, unlike all other networks that show higher response in the former.

**Figure 6:**
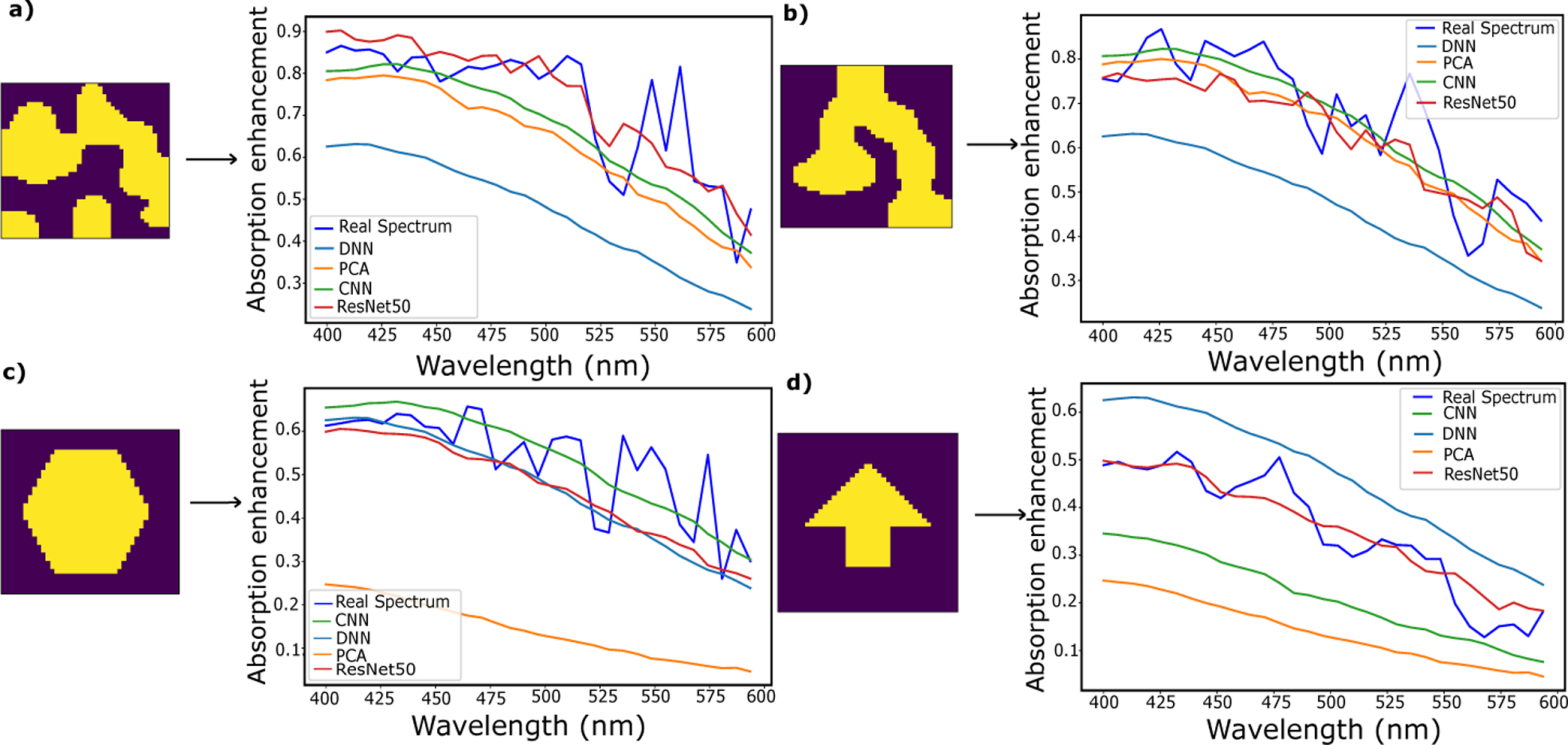
(ab) Two example interpolative structures and the predicted spectra of the forward networks. (cd) Two example extrapolative structures and the predicted spectra of forward networks.

To further understand the differences between the responses of the various forward network types, we compare the full label spectra, input structure, and predicted response by the four systems, as shown in Figure 6. Two representative samples from the randomly produced structures in the validation set are shown in Figure 6a,b, along with two representative structures from the extrapolation set in Figure 6c,d. Extrapolation set samples show numerous features that are absent from the training set, including sharp features, right angles, and varying degrees of symmetry. It is again clear that in interpolation, the DNN significantly underpredicts the entire spectrum of the structures, as well as showing none of the corresponding peaks and troughs in the true label response. Figure 6d is perhaps the most telling demonstration of the higher accuracy of the ResNet50 architecture, showing a strong correlation to the true label spectrum, while the other three networks significantly overestimate (DNN) or underestimate (PCA and CNN) the actual response. This suggests that the use of a network trained on an application vastly different from the one being investigated (such as the photographic image recognition training of ResNet50) can have benefits in avoiding bias toward features present in only the training set.

### Inverse Modeling Results

After exploring the creation and testing of the forward networks, we next investigate the performance of inverse networks to generate structures from a desired input spectrum. The ability to generate structures with specific properties is central to the design of high-performance nanophotonic structures. Furthermore, if ML systems are able to extrapolate significantly beyond the bounds of the training configuration space, such approaches could be used to further enhance performance even for structures already designed through local topology optimization processes. To test this, we implement five different networks and compare their performance for both interpolative and extrapolative spectra.

**Figure 7:**
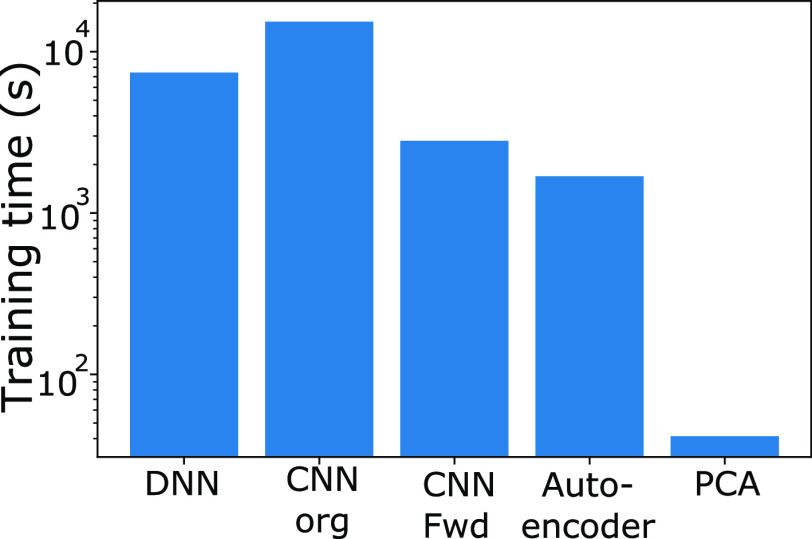
Total time taken for training and convergence of the inverse networks—DNN; CNN utilizing the original (label) spectra (CNN Org); CNN filtering the label spectra through the forward CNN (CNN Fwd); autoencoder combined with the DNN; and the inverse PCA combined with the DNN (PCA, including PCA training time).

The plot of the total time taken to converge the inverse networks is demonstrated in Figure 7. Comparison of validation loss between these networks during the training cycle is not useful due to different loss functions being used for the different networks. However, the total time to train such networks is indeed still comparable. In contrast to the forward networks, here four of the five inverse networks require the same order of magnitude of time for training, with one, the PCA + DNN approach, taking a significantly shorter time. Interestingly, the time to run the reverse PCA process is insignificant, requiring less than 1 s. The reduced complexity of the network, thanks to the PCA dimensionality reduction, is again able to significantly reduce the training time, here to less than 1% of that needed to train the pure DNN.

Similar to the forward networks, the performances of inverse networks were also characterized based on two metrics: the MSE between the expected spectrum and the spectrum obtained from the generated structures (M1), as well as the minimum of the maximum difference between peaks of spectra, considering the subset produced using a fixed input spectra but with a range of input noise vectors (M2). M1 thus represents the ability on average for a system to produce structures that match a target spectrum, while M2 indicates the ability of a system to produce at least one high-quality structure through the use of different noise inputs to replicate the desired response, including all peaks and troughs of the spectrum. Both metrics are investigated as either metric may be more useful for a particular design problem: for some applications, a high consistency across all designs may be useful (e.g., in reconfigurable optics, where topologies are modified to match an arbitrary response), while in other applications, just being able to generate one very high-accuracy response (or one response with novel features), particularly when the results will be checked by simulations after, is more valuable.

**Figure 8:**
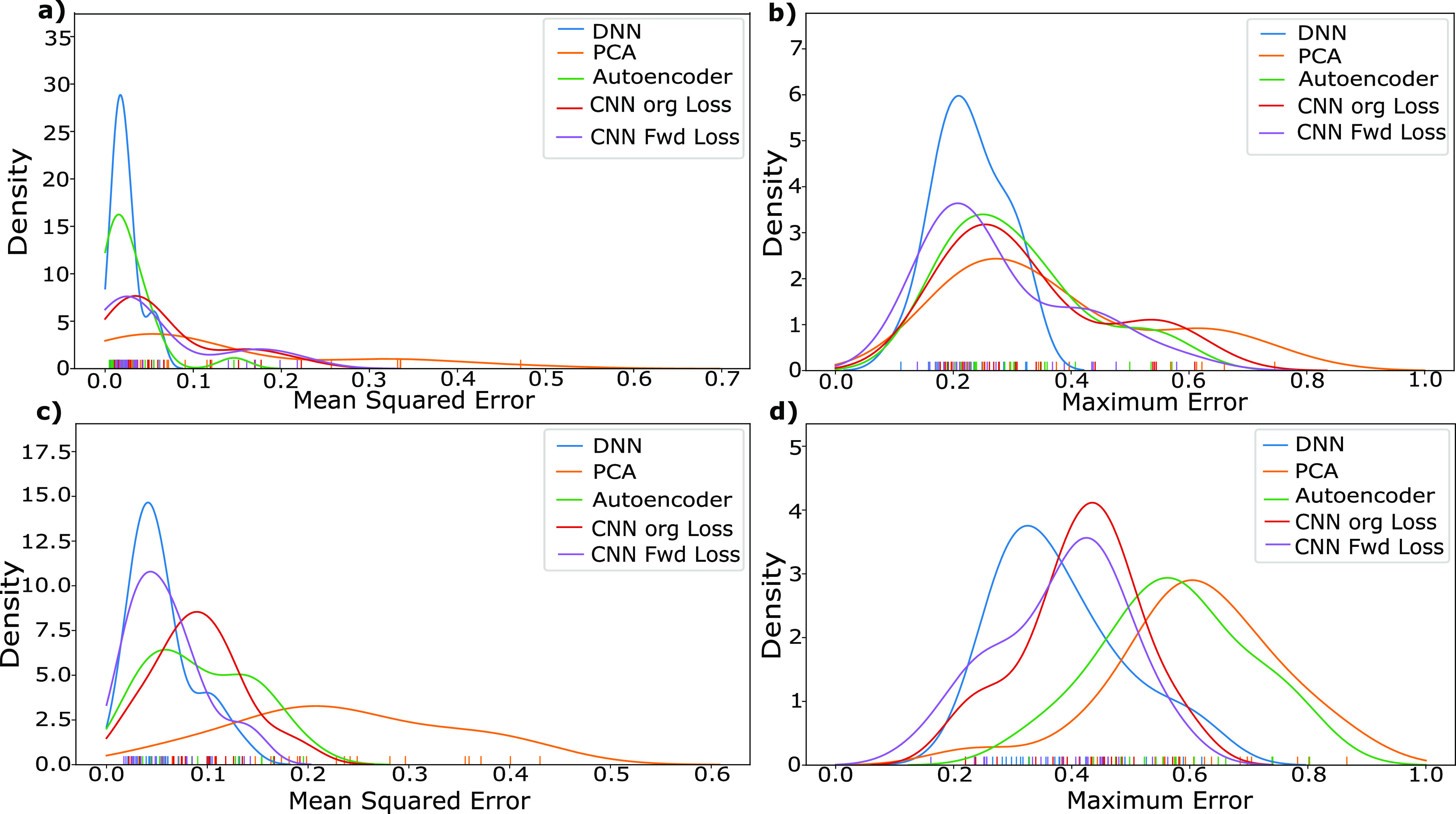
MSE between the spectra of the generated structures and the input spectra (M1) for interpolation (a) and extrapolation (c). Minimum of maximum difference between the expected and obtained spectra across 10 structures generated from 10 different input noise vectors (M2), for interpolation (b) and extrapolation (d). Color tick marks indicate the location of the data points.

Figure 8 demonstrates the variation of mean performance (M1) and ability to produce a sample with good peak/trough prediction (M2) for both interpolative and extrapolative spectra for all five networks. Although it was seen that the DNN performed worst as a forward network, it now performs quite well as an inverse network in both interpolation and extrapolation, showing the lowest error of any of the considered approaches. We believe that this may be due to more specialized networks (i.e., those that include convolutional layers and PCA) attempting to extract important features and predict the results based on these learned features, with the assumption of some invariance to either displacements or rotation. This however may break down in the inverse network, in which a system may try to place a feature it has seen correlate to a particular response in a position (relative to other features) or orientation that no longer would lead to a similar response. Conversely, as a forward network, the DNN does not have specific architectural components to detect features but simply learns correlations between input pixels to find the mapping between the structure and spectrum. There may thus be too much structural diversity in the forward network to allow the DNN to perform well; however, as an inverse system, it needs only be able to generate one type of structure which can match an input spectrum, as opposed to knowing the result of any arbitrary structural input.

When investigating the two CNNs implemented here, another interesting correlation is observed—the network that utilizes the approximate response by sending the label structure through a forward CNN shows higher performance and a shorter training time than the CNN utilizing the original (real) label spectral response. This suggests that giving the inverse network a potentially easier task during training—that is, learning the response expected by a forward CNN, as opposed to the need to learn the true physical response—actually makes the system better at producing structures with correct physical spectral responses after training.

Finally, we note that although a combined approach of PCA + DNN showed better performance than the pure DNN in forward interpolation, it does not perform well as an inverse network. Here, the autoencoder displays a more comparable response to the PCA + DNN process in forward modeling, in which it shows relatively high performances in both M1 and M2 for interpolation but performs significantly worse in extrapolation.

**Figure 9:**
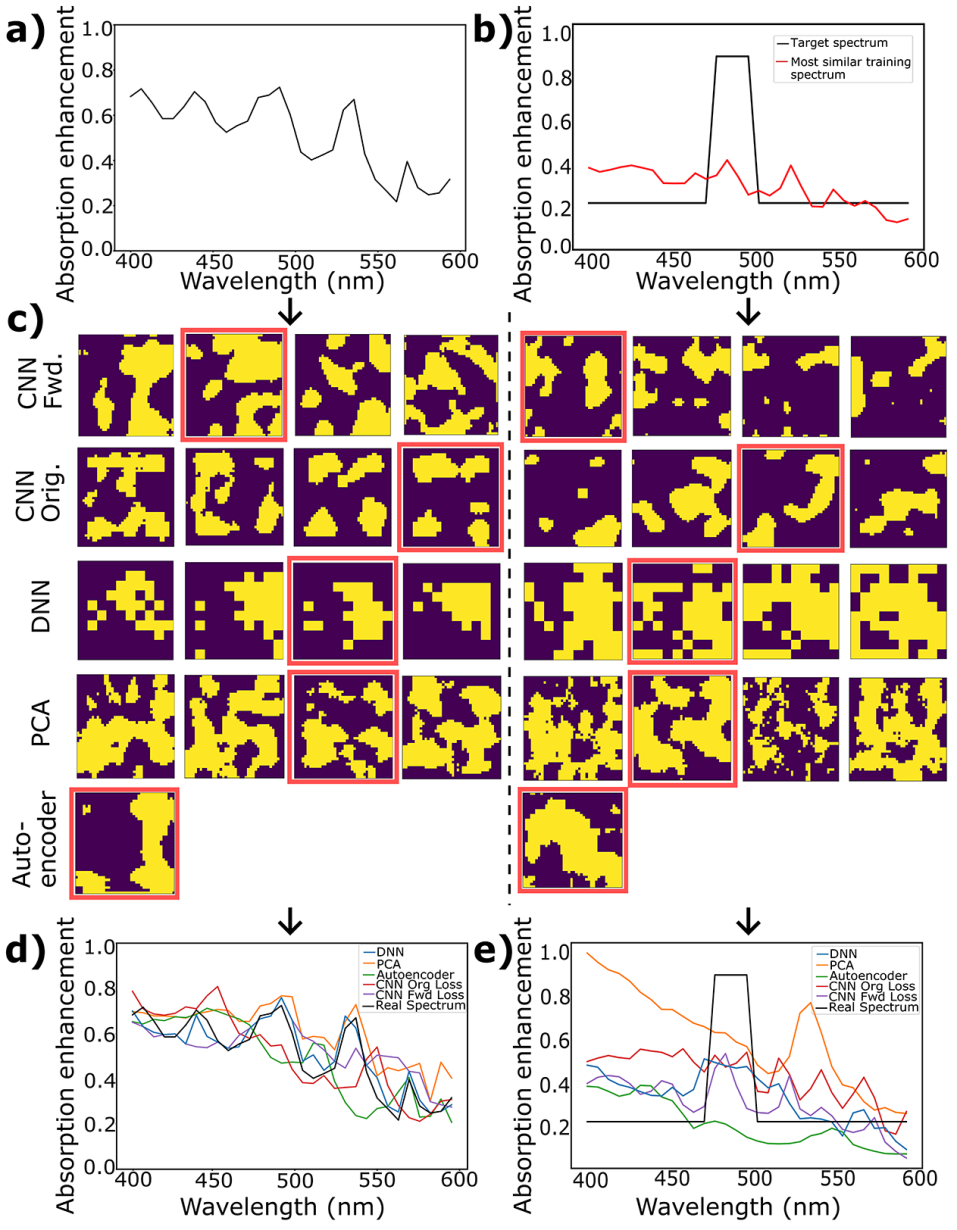
Generated images from inverse networks. (a) Representative interpolative input spectrum. (b) Extrapolative input spectrum (black) along with the most similar spectrum in the training set (red), confirming that this spectrum is indeed significantly different from those in the training set. (c) Structures generated by the five systems for both of the input spectra shown above for four different noise vectors. (d) Spectra for generated interpolative structures and (e) spectra for generated extrapolative structures, as determined by FDTD simulations of the samples above with the lowest-error structure (highlighted in red) for each.

To understand the generation capability of inverse networks more clearly, we again explore specific representative samples, both in interpolation (Figure 9a,c,d) and extrapolation (Figure 9b,c,e). At the top of the figure, the two input spectra are shown, and in the middle, the corresponding structures are produced by taking different noise vectors with each investigated network. The highlighted structure of each network is the structure with the spectrum shown in the bottom plot and corresponds to that with the lowest loss of the four options. To find the best-generated structure closest to the target spectrum, the loss values were calculated based on the spectra obtained after running the generated structures through FDTD simulations. We should note that in extrapolation particularly, the input spectrum is highly idealized and more meant to represent a general type of behavior being tested than an actual anticipated performance. For example, in the spectrum shown here, an increased absorption efficiency in a particular band, from 475 to 500 nm, is requested, but it is unlikely that there is any physical structure within the potential configuration space that would produce such an idealized (selective) response. In this sense, the DNN output is actually surprisingly reasonable, showing the requested absorption enhancement in the correct spectral location and a decreased response elsewhere. This is especially impressive when compared to the closest example from the entire training set, shown as the red curve in Figure 9b, indicating that this network is indeed able to extrapolate responses significantly different from those on which it is trained.

The relatively high accuracy of the DNN response, as well as the corresponding structural shape further confirms the results shown in Figure 8 while helping to explain the observed performances. The generated structures from the DNN are combinations of small squares but with sizes significantly larger than the matrix pixel size. This indicates that the DNN was able to reduce the complexity of the output space by lowering the effective resolution while still utilizing it accurately (indeed, even more accurately than the other networks). While showing the highest performance here, this reduced complexity may limit the ability of the network to produce some potential features. This limitation however was not observed in the present study.

Additional comparisons between input extrapolation spectra and output spectra from generated structures are shown in Figure S5 of the Supporting Information. These further support that the DNN performs relatively better compared to other networks in generating structures having expected absorption enhancement features. The extrapolation performance of the CNNs is also seen to be performing comparatively better than ensemble inverse PCA + DNN and autoencoder + DNN.

## Conclusions

In this work, we explore a range of diverse prototypical ML techniques for both forward modeling and inverse design of nanophotonic structures. The comparison between the interpolation and extrapolation sets indicates that different networks show different relative performances in interpolation and extrapolation, with good performance in one not necessarily correlated with high performance in the other. A CNN and ensemble approach of PCA + DNN has a lower loss compared to a pure DNN approach. The benefits of adding PCA to the DNN interestingly partially confirm that best performances are obtained from combined ML and ANN techniques, for which they work well for forward interpolation in terms of lower loss and lower computational time, although they show lower performance at extrapolation. In general, a ResNet50 pretrained network shows the highest performance in both interpolation and extrapolation for forward prediction. This suggests that even when trained on entirely unrelated tasks (e.g., image classification), pretrained networks may still provide significant benefits to new applications.

Furthermore, we show that while a pure DNN exhibits fairly low performance as a forward network, it shows the highest performance as a generative inverse network. This network also shows remarkably high performance at extrapolation, with the ability to produce structures with spectra significantly different from those it has been trained upon. This is somewhat surprising as such networks are rarely used in generative tasks of image-like outputs, likely due to an assumption that higher-complexity CNNs would perform better due to their abilities in feature abstraction. With further parameter and architecture tuning, and potentially iterative training, such networks may be able to significantly expand the configuration space for nanophotonic design.

One of the significant benefits of using ML and ANN techniques over conventional approaches is that once the networks are trained, the predictions or generation of structures can be performed in a fraction of a second. This, in particular when combined with extrapolation abilities, can permit new types of structures with continually varying properties and reconfigurable geometries, which would not be feasible otherwise.

To further improve the performances of the networks investigated here, a range of additional techniques can be further implemented. Particularly, iterative training techniques^28,31^ have been introduced in several recent works, where the training set expands after one training cycle to improve the performances of failing samples. While outside the scope of the work presented here, these and other post-training approaches could improve the forward and inverse response quality of similar networks.

While this work has focused on a simple 2D nanoparticle design, the general approach and key results can be further extended to predict other optical properties and generate structures corresponding to other desired optical responses for both 2D and 3D configurations. The investigated 2D simulations correspond to structures being extended indefinitely in the third dimension. Fully 3D-structured particles would likely require more complex networks and may yield different relative responses. Investigation into the use of similar networks for other types of 3D structures is ongoing.

## Supporting Information Available

Data preparation, simulation setup, and convergence; loss functions used in network training; forward and inverse network architecture; and additional examples of generative design results (PDF)
