# Supplementary material for: Machine Learning in Interpolation and Extrapolation for Nanophotonic Inverse Design
Source: ACS Omega. 2022 Sep 9;7(37):33537–47. doi: 10.1021/acsomega.2c04526 (PMC9494689; doi:10.1021/acsomega.2c04526)
Supplement: Supplementary file 1 — ao2c04526_si_001.pdf [file ao2c04526_si_001.pdf]

Supporting Information for Machine Learning in Interpolation and Extrapolation for Nanophotonic Inverse Design

Didulani Acharige* and Eric Johlin*

Department of Mechanical and Materials Engineering, Western University, London, ON

E-mail: dsalwath@uwo.ca; ejohlin@uwo.ca

Data Preparation

We generated 10,000 2D random structures to train the deep networks. In order to do that first, we generated random structures by adding random rectangular features to a 2D array with 40 × 40 resolution, and smoothing the resulting array. These arrays were used to dictate the presence or absence of silicon in a $500nm \times 500nm$ square bounding region. Simulations were conducted to obtain the absorption enhancement values using the open-source finite-difference time-domain (FDTD) simulation software Meep.[1] This process is shown schematically in Fig. S1.

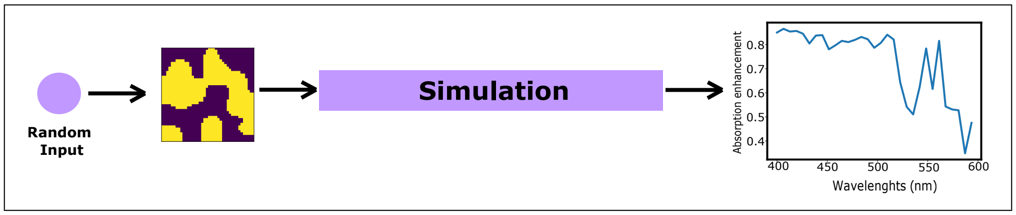

Figure S1: Schematic of training set creation, showing randomization of structures, FDTD simulation and the respective absorption spectrum output.

For the simulations, a plane wave with wavelengths from 400 nm to 600 nm is launched towards the structure in the +x-direction. The full simulation region is 2,000 nm, with 400 nm thick perfectly matched layer (PML) boundaries surrounding the simulated area.

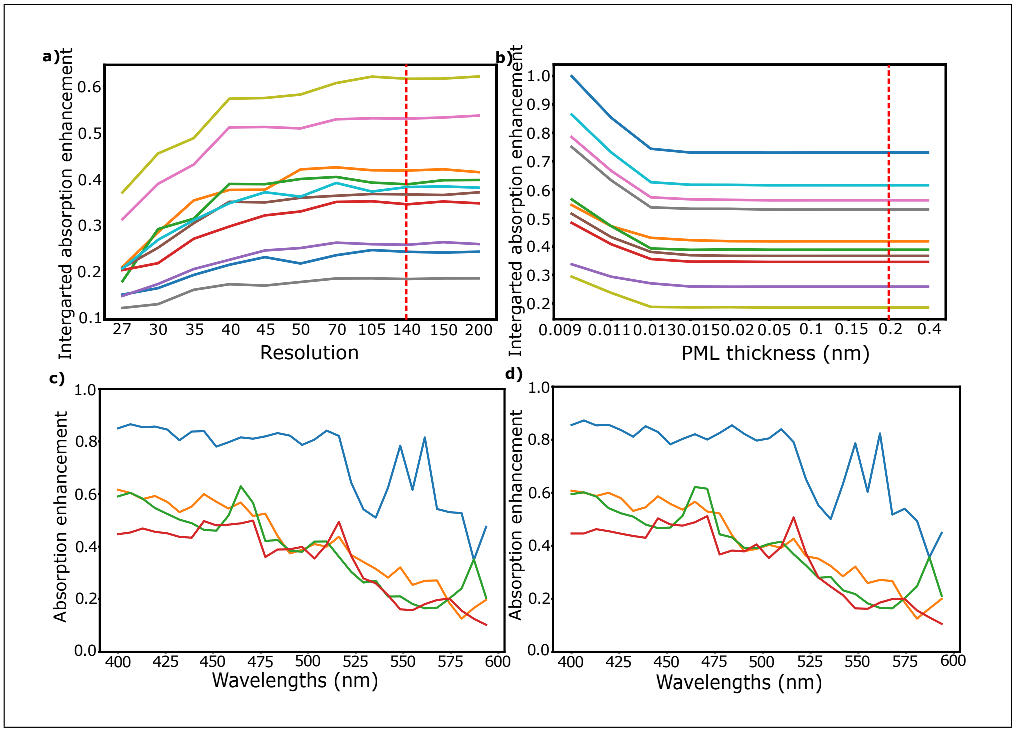

Figure S2: Convergence plots showing the mean integrated absorption enhancement for (a) varying resolutions (number of pixels per micron), and (b) varying PML thicknesses, each for 10 representative structures. (c) Four different spectrums related to structures at 140 resolution (d) Same spectrums at 200 resolution

Simulation convergence is depicted in Fig. S2. Specifically, Fig. S2a shows the integrated absorption relating to different resolutions for ten representative samples. Moreover, the integrated absorption resultant from different PML thicknesses is shown in Fig. S2b. The red dashed lines represent the parameters used to obtain the absorption enhancements for the randomly generated structures from simulations. Further, Fig. S2c and Fig. S2d visualize a few of the full spectra for the 140 and 200 spatial resolutions respectively, where 140 is the spatial resolution we utilized for our simulations and 200 is the highest resolution we explored to confirm that the spectrums are clearly remain unchanged as the simulations are properly converged.

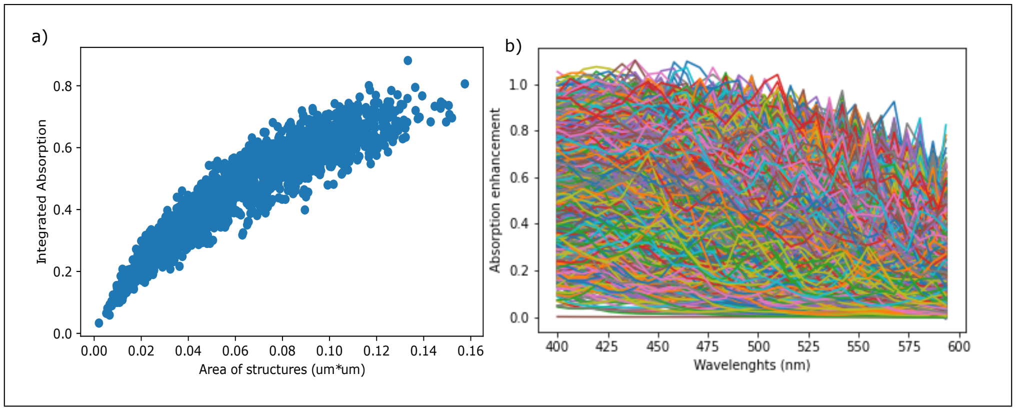

Figure S3: (a) Mean absorption enhancement with respect to the area of material present on the structures for the entire test ensemble, showing the variety of responses, as well as impact of structure present. (b) Absorption enhancement spectra corresponding to the full training dataset, showing the range and general scope of data used for training.

The diversity of nanophotonic responses is shown in Fig. S3a, demonstrating that the calculated integrated absorption depends not only on the amount of material present, but also strongly on the configuration of the structures. This is visible as the spread in the mean absorption away from a single line, as different configurations with the same amount of material show significantly varied optical response. The obtained absorption enhancement spectra corresponding to the generated structures are given in the Fig. S3b While there are clearly too many spectra to distinguish most individual trends, the overall ensemble performance is clear, with absorption enhancements ranging from 0 to 1.1, and generally larger values at smaller wavelengths, due to the absorption spectrum of silicon.

After obtaining the absorption enhancements corresponding to each structure the data set was split as training and testing data for the network training and validation. Among the total 10,000 original data, 80% of the data are used for training, and the remaining 20% was used as testing data.

Loss Functions

Traditional machine learning algorithms and ANNs are trained using an optimization process that involves a loss function to calculate the model error, with the goal of the optimization being to minimize said error. Therefore, as part of the optimization process, it requires repeatedly estimating the error for the current state of the model, so that the weights of each neuron can be updated in order to decrease the loss on the following round of optimization. Here, we are concerned with a regression task, as our goal is to allow the machine learning systems to predict continuously valued spectral responses of silicon nanoparticles to optical excitation. These type of predictive modeling problems require predicting a real-valued scalar quantity. In this work we used one of the three different loss functions shown below for the investigated forward and inverse networks, with the choice dictated by the presence or absence of input data.

The first function, $Loss_1$ calculates the mean squared error between the real and the prediction functions, while $Loss_2$ represents the binomial cross-entropy function. The final function, $Loss_3$, is a customized loss function made from $Loss_1$ and $Loss_2$, similar to that introduced by So. S et. al in their work to train cDCGAN.[2] The first loss function can be represented as

$$Loss_1 = \frac{1}{n}\sum_{i=1}^{n}(x_i - \bar{x}_i), \tag{S1}$$

where n is the number of training or testing samples and xi and $\bar{x}_i$ are corresponding label value and the predicted value, respectively. The second loss represents the similarity between two structural matrices, calculated as

$$Loss_2 = \frac{1}{N}\sum_{i=1}^{N} -(y_i \log(p_i) + (1 - yi)\log(1 - p_i)), \tag{S2}$$

where N is the number of samples, y is the label value for one point in the matrix, with 1 representing presence of material, and 0 the absence. The term p_i is the network's predicted

probability of the point being 1 (*i.e.* contains material), whereas $(1 - p_i)$ representing the predicted probability of a point being 0 (does not consist material). The final function simply combines these with

$$Loss_3 = (\alpha \times Loss_2) + (\beta \times Loss_1) \tag{S3}$$

and the parameters α and β simply representing weighting terms for the two sub-components of the overall loss function.

The loss functions of all networks were optimized using the Adaptive Moment Estimation (Adam) optimizer algorithm. When using customized loss (Eq. S3) for inverse networks α and β parameters were chosen based on analyzing the results obtained from changing the parameters accordance with trial-and-error approach to dominate the mean squared error between real spectra and the spectra of the generated structures.

Forward networks

The input and the output nodes of all networks are adjusted to match the structure size (1,600 nodes) and the spectrum size (31 nodes). The weights of the internal layers of the pre-trained ResNet50 network were implemented as pre-trained weights publicly available from ImageNet.[3,4] The pure DNN network was implemented as having the same number of input and output nodes, and 5 hidden layers, with 1024,1024,512,256,128 nodes within these layers.

We use the TensorFlow framework and Keras back-end for the deep learning algorithms and the networks were optimized using Adam optimizer. The network architectures and details about four different forward networks are given below.

ResNet50 pre-trained network

ResNet50 is a convolutional neural network, and it is 50 layers deep. In this work, we have loaded a pre-trained version of this network which was trained on more than a million images

from the ImageNet database. The number of input nodes and the output nodes was changed to the structure resolution and number of wavelengths in the spectra respectively.

Deep Neural Network

The simplest DNN was implemented with four hidden layers and the number of input nodes and output nodes was the number of pixels in the structure and the number of wavelengths in the spectrum respectively. The network architecture along with the hyperparameters used are given in Tab. S1 below. The deep layers of this network were kept unchanged in CNN and an ensemble approach of PCA and DNN make the comparisons more effective.

Convolutional Neural Network

For the CNN the structure images were fed as inputs and the number of output nodes was the number of wavelengths in the absorption enhancement spectra. CNN networks are promising to extract important features from the images from the convolutional process. The hyperparameters used for the network are given in Tab. S1 below.

An ensemble approach of Principal Component Analysis (PCA) and Deep Neural Network (DNN)

Large datasets are usually difficult to interpret. PCA is a technique to reduce the dimensionality of data by extracting the most dominant features to increase the interpretability of data at the same time minimizing the information loss. It is a process of calculating the principal components and utilizing them to perform a change of basis on the data to reduce the dimensionality. Since the ensemble approaches are promising to improve the performances, in our work we suggest decreasing the dimensionality of structural images and feeding the new data to a DNN to find the mapping to predict the optical response. Fig. S4A demonstrates the cumulative explained variance plot and that implies even with 500 components we can

represent over 95% of data. Further, the original structure images and constructed images with 50 and 500 components are given in Fig. S4B. That further confirms the results we obtained from Fig. S4A.The hyperparameters used for the network is given in the Tab. S1.

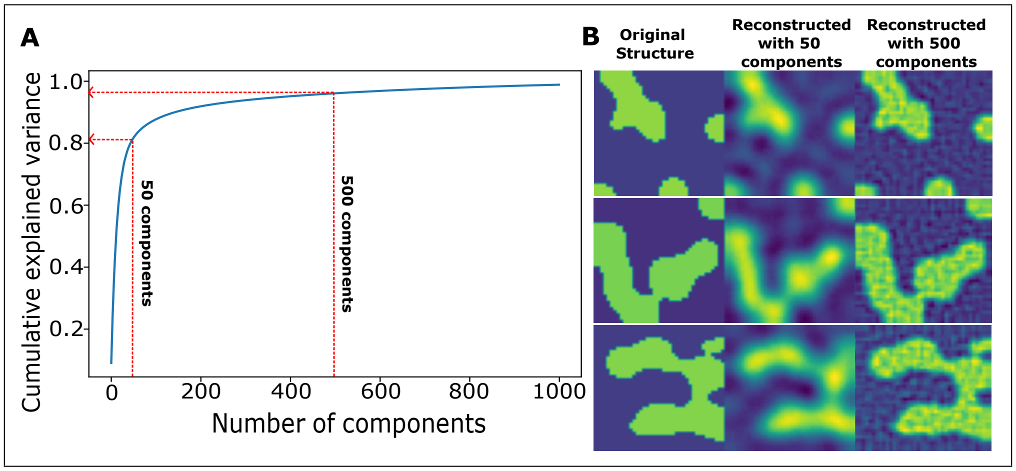

Figure S4: (a) Cumulative Explained Variance over principal components (b) Three different original structures and reconstructed structures with 50 and 500 principal components respectively

Table S1: Different Architectures of forward networks with hyperparameters

H	DNN	CNN	PCA and DNN
HL	5	5	5
HN	1024, 1024, 512, 256, 128	1024, 1024, 512, 256, 128	1024, 1024, 512, 256, 128
DP	0.5,0.25, 0.4, 0.5	0.5,0.25, 0.4, 0.5	0.5,0.25, 0.4, 0.5
NCL	-	2	-
NCCL	-	64,64	-
PT	-	AveragePooling2D	-
DPCL	-	0.3, 0.3	-
KS	-	(3,3)	-
AF	ReLU, Linear	ReLU, Linear	ReLU, Linear
LF	MSE	MSE	MSE

H - Hyperparameters

HL - Number of hidden layers

HN - Number of nodes in each hidden layer

DP - Dropout percentages in each layer

NCL - Number of convolutional layers

NCCL - Number of channels in the convolutional layers

PT - Pooling type

DPCL - Dropout percentages in convolutional layers

KS - Kernel Size

AF - Activation functions

LF - Loss function

Inverse networks

As our first generative network, we implemented a DNN to generate structures from the desired input spectra. A customized loss is given in Eq. S3 with α as 0.4 and β as 0.6 had been used to train the network. These values are chosen to increase the weight of the actual performance of the network, while still allowing the inclusion of comparison between label and predicted structures, which are particularly important in the early training of the weights. When calculating the loss values to optimize networks, the spectrum loss (second component of $Loss_3$) was calculated based on the expected spectra and the spectra obtained for generated images from implemented pre-trained CNN network. Note that the weights of the pre-trained forward CNN network are frozen. As the second and third inverse networks, we implemented two CNN networks with up-sampling layers. In the first CNN network, we used the Loss 3 function from Eq. S3 where the second component was calculated based on the label values of the spectra compared to the predicted performance of the generated structure. In the second network, the second component of the loss was calculated considering the spectra related to generated structures and the spectra received after feeding the structures to the best performing CNN forward network. In both CNN networks, a noise vector scaled in 0 and 0.1 is fed into the network to generate different spectra for similar absorption enhancement spectra. The α and β values for the networks were taken as the same values

as for the DNN network loss function.

In the fourth network, the structures generated from CNN were encoded to a latent space, and then it is fed to a decoder network to match the structures to the original structures. The autoencoder network was trained based on the binary cross-entropy (Eq. S2) loss, as its purpose was to produce a structure based on lower-dimensional input features, as opposed to a known spectral input. Finally, as a combined approach an inverse PCA method was implemented by feeding the 500 components obtained from DNN to the inverse PCA algorithm to generate structures. The PCA network that was previously trained during the forward modelling was used here, simply with a reversed propagation through the system.

We use the TensorFlow framework and Keras backend for the inverse deep learning algorithms and the sequential networks were optimized using Adam optimizer. The network architectures and details about five different inverse networks are given in the Tab. S2.

The generated images from inverse networks were binarized using a binarization function. The threshold value was calculated considering the median of the set of the generated images. The generated structures were further simulated on MEEP to find their real spectra. The performances of the networks were analyzed based on the expected spectrum and the spectra of the generated structures.

Table S2: Different Architectures of five different inverse networks with hyperparameters

H	DNN	CNN org	CNN fwd	PCA and DNN	Autoencoder
NHL	3	-	-	1	-
NNHL	256, 128, 64	-	-	750	-
DPL	0.6, 0.6, 0.6	-	-	0.5	-
NCL	-	4	4	-	5
NCCL	-	256, 128, 64, 1	256, 128, 64, 1	-	32, 32, 32, 32, 1
PT	-	-	-	-	AveragePooling
DPCL	-	0.6, 0.6, 0.6	0.6, 0.6, 0.6	-	-
KS	-	(3,3)	(3,3)	-	(3,3)
NUL	2	2	2	-	2
AF	ReLU, LeakyReLU	LeakyReLU	LeakyReLU	ReLU, Sigmoid	ReLU
LF	CL	CL	CL	MSE	BCE

H - Hyperparameters

NHL - Number of hidden layers

NNHL - Number of nodes in each hidden layer

DPL - Dropout percentages in each layer

NCL - Number of convolutional layers

NCCL - Number of channels in the convolutional layers

PT - Pooling type

DPCL - Dropout percentages in convolutional layers

KS - Kernel Size

NUL - Number of Upsampling layers

AF - Activation functions

LF - Loss function

CL - Customized loss ($\alpha = 0.4$, $\beta = 0.6$)

BCE - Binary Crossentropy

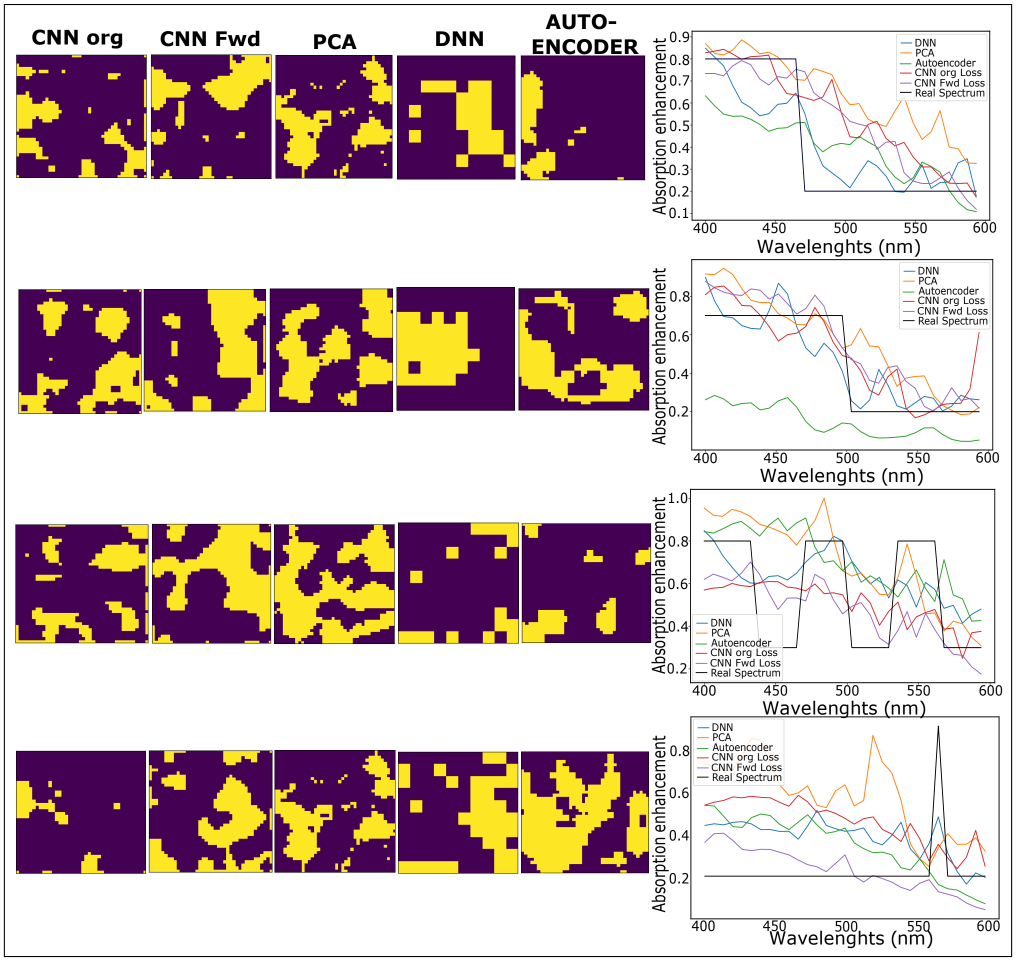

Figure S5: Generated structures with inverse networks, expected spectra and spectra related to the generated structures

Fig. S5 depicts a representative structures generated by each of the five generative networks for four sample extrapolation spectra, and the comparison of the true spectra for the generated structures. Even though the requested extrapolation spectra are not expected to be physically possible to match, the goal here is to determine how far the network can push the spectra toward extreme and novel responses. Here, we can see that DNN generally performs comparatively better in generating a structure that includes some features of the requested extrapolation spectrum than the other approaches.

References

(1) Peng, Y. Simulations of optical effects in nanostructures. Ph.D. thesis, Boston College, 2011.

(2) So, S.; Rho, J. Designing nanophotonic structures using conditional deep convolutional generative adversarial networks. *Nanophotonics* **2019**, *8*, 1255–1261.

(3) He, K.; Zhang, X.; Ren, S.; Sun, J. Deep residual learning for image recognition. Proceedings of the IEEE conference on computer vision and pattern recognition. 2016; pp 770–778.

(4) Akiba, T.; Suzuki, S.; Fukuda, K. Extremely large minibatch sgd: Training resnet-50 on imagenet in 15 minutes. *arXiv preprint arXiv:1711.04325* **2017**,
